# 

**Published:** 1892-01-23

**Authors:** 


PRICE ONE PENNY.

No. 278, Vol. XI.—January 23rd, 1892.

[Registered at the General Post Office as a Newspaper for transmission in the United Kingdom and Abroad.]

WITH EXTRA NURSING SUPPLEMENT.
Per Annum, 6s. 6d., post free.
Monthly Parts, 6d.; post free, 8d.
Ditto per Annum, 8s., post free.

### THE HOSPITAL

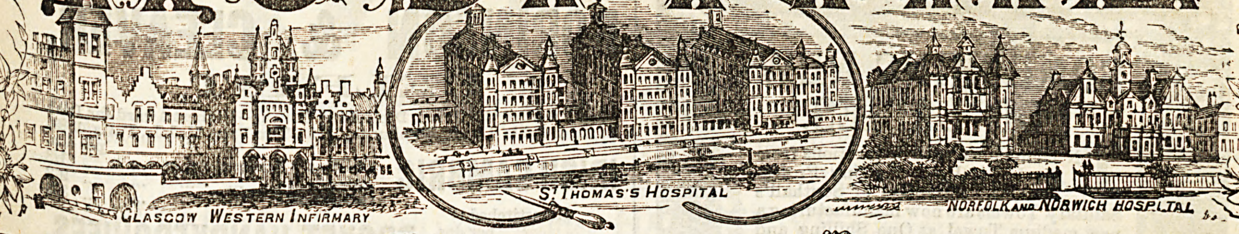

A WEEKLY JOURNAL OF

### Science, Medicine, Nursing, and Philanthropy.

SATURDAY, JANUARY 23rd, 1892.

**The Nurses' Tribute to the Dead Prince** - 201
**Passing Topics.**—The Ordeal of Burial - 202
**The Doctor's Armchair.**—Are all Writers Men of Genius? - 203
**Some Scientific Aspects of Insanity.** IX.—Attention due to Maniacs - 204
**Some American Hospitals.** IV.—Denver - 205
**Everybody's Page** - 205
**Notes and News** - 206
**General Charities—Scraps and Gleanings** - 207
**Annotations.**—Cardinal Manning—A Famous Cancer Cure—The Influenza Microbe - 208
**The Practitioner's Mirror and Retrospect**—
Medicine.—Blepharospasm—On Some Urine Tests. II. - 209
Practitioner's Bookshelf.—Inherited Consumption, 210; Health in Schools - 211
**The Story of the Insane from Year to Year** - 212
**The Student of Medicine.**—Appointments—Vacancies—Notes from the Schools—Pass Lists.

EXTRA SUPPLEMENT.—THE NURSING MIRROR.

**En Passant.**—An Expression of Sympathy—Starting District Nurses—The late Duke of Clarence—Frome Nurses' Home—The Religious Question—The Nurses' Co-operation xcvii

**Lectures on Surgical Ward Work and Nursing.** Lecture XLII.—Instruments used in Lithotomy - xcviii

**For Reading to the Sick.**—Bardens - xcix

**Keeping Christmas** - c

**District Nurses for Brighton and Hove.** By the Hon. Roden Noel - c

**On the Nursing of Children.** II.—Emergencies - ci

**Hints for Nurses** - ci

**Everybody's Opinion.**—Amateurs on Nursing - ci

**Off Duty.**—Those Three - cii

**Presentations—Appointment—Notes and Queries** - cii

